# Characterizing HIV-1 Genetic Subtypes and Drug Resistance Mutations among Children, Adolescents and Pregnant Women in Sierra Leone

**DOI:** 10.3390/genes12091314

**Published:** 2021-08-26

**Authors:** George A. Yendewa, Sulaiman Lakoh, Sahr A. Yendewa, Khadijah Bangura, Andrés Tabernilla, Lucia Patiño, Darlinda F. Jiba, Alren O. Vandy, Samuel P. Massaquoi, Nuno S. Osório, Gibrilla F. Deen, Foday Sahr, Robert A. Salata, Eva Poveda

**Affiliations:** 1 Department of Medicine, Case Western Reserve University School of Medicine, Cleveland, OH 44106, USA; ras@uhhospitals.org; 2 Division of Infectious Diseases and HIV Medicine, University Hospitals Cleveland Medical Center, Cleveland, OH 44106, USA; 3 Johns Hopkins Bloomberg School of Public Health, Baltimore, MD 21205, USA; 4 Department of Medicine, College of Medicine and Allied Health Sciences, University of Sierra Leone, Freetown, Sierra Leone; lakoh2009@gmail.com (S.L.); syendewa@gmail.com (S.A.Y.); gibrilladeen1960@yahoo.com (G.F.D.); fsahr65@gmail.com (F.S.); 5 Ministry of Health and Sanitation, University of Sierra Leone Teaching Hospitals Complex, Freetown, Sierra Leone; khadijahbangura83@gmail.com (K.B.); darlindajiba.dj@gmail.com (D.F.J.); alrenvandy@gmail.com (A.O.V.); drspem@gmail.com (S.P.M.); 6 Group of Virology and Pathogenesis, Galicia Sur Health Research Institute (IIS Galicia Sur), Complexo Hospitalario Universitario de Vigo, SERGAS-UVigo, 36213 Vigo, Spain; andrestabernilla@hotmail.com (A.T.); luciapatinoalvarez@gmail.com (L.P.); eva.poveda.lopez@sergas.es (E.P.); 7 Life and Health Sciences Research Institute (ICVS), School of Medicine, University of Minho, 4710-057 Braga, Portugal; nosorio@med.uminho.pt

**Keywords:** HIV, drug resistance, children, adolescents, pregnant women, resource-limited settings, Sierra Leone

## Abstract

Human immunodeficiency virus (HIV) drug resistance (HIVDR) is widespread in sub-Saharan Africa. Children and pregnant women are particularly vulnerable, and laboratory testing capacity remains limited. We, therefore, used a cross-sectional design and convenience sampling to characterize HIV subtypes and resistance-associated mutations (RAMs) in these groups in Sierra Leone. In total, 96 children (age 2–9 years, 100% ART-experienced), 47 adolescents (age 10–18 years, 100% ART-experienced), and 54 pregnant women (>18 years, 72% ART-experienced) were enrolled. Median treatment durations were 36, 84, and 3 months, respectively, while the sequencing success rates were 45%, 70%, and 59%, respectively, among children, adolescents, and pregnant women. Overall, the predominant HIV-1 subtype was CRF02_AG (87.9%, 95/108), with minority variants constituting 12%. Among children and adolescents, the most common RAMs were M184V (76.6%, *n* = 49/64), K103N (45.3%, *n* = 29/64), Y181C/V/I (28.1%, *n* = 18/64), T215F/Y (25.0%, *n* = 16/64), and V108I (18.8%, *n* = 12/64). Among pregnant women, the most frequent RAMs were K103N (20.6%, *n* = 7/34), M184V (11.8%, *n* = 4/34), Y181C/V/I (5.9%, *n* = 2/34), P225H (8.8%, *n* = 3/34), and K219N/E/Q/R (5.9%, *n* = 2/34). Protease and integrase inhibitor-RAMs were relatively few or absent. Based on the genotype susceptibility score distributions, 73%, 88%, and 14% of children, adolescents, and pregnant women, respectively, were not susceptible to all three drug components of the WHO preferred first-line regimens per 2018 guidelines. These findings suggest that routine HIVDR surveillance and access to better ART choices may improve treatment outcomes in Sierra Leone.

## 1. Introduction

According to the World Health Organization (WHO) 2019 report on HIV drug resistance (HIVDR), the emergence and spread of HIVDR in low- and middle-income countries (LMICs) was a major factor limiting the success of antiretroviral treatment (ART) programs in achieving the 90–90–90 global targets for 2020 [1]. The situation was most dire in sub-Saharan Africa (SSA), which recorded the highest rates of HIVDR [1]. The WHO report further revealed that children and women are particularly vulnerable, with the prevalence of pre-treatment HIVDR to non-nucleos(t)ide reverse transcriptase inhibitors (NNRTIs) greater than 50% in children aged <18 months, while overall, women were twice as likely as men to harbor HIVDR mutations [1]. In response to the increasing rates of HIVDR globally, a five-year Global Action Plan on HIVDR (2017–2021) was rolled out, which called for the integration of HIVDR monitoring into broader HIV prevention and control efforts [2]. However, with recent disruptions in HIV care resulting from the COVID-19 pandemic, the overall impact of this global agenda remains uncertain.

Sierra Leone is a low-income West African country of 7.4 million people. The HIV epidemic in the country is described as generalized, with an estimated national HIV prevalence of 1.5% in 2018, i.e., 70,000 people living with HIV, of whom 6600 were children aged 0–14 years [3]. The National AIDS response of Sierra Leone was first launched in 2002—shortly after the country emerged from an 11-year period of civil warfare (1991–2002)—to help combat the growing HIV epidemic. Following the Ebola epidemic of 2014–2016, which led to severe disruptions in HIV service delivery in the country [4,5,6,7], the Government of Sierra Leone announced a five-year National Strategic Plan (NSP) on HIV/AIDS, which aimed to increase routine HIV testing activities, bolster the prevention of mother-to-child transmission (PMTCT) services, and expand ART coverage [8]. The UNAIDS 2019 report revealed that from 2010 to 2018, there was a 27% decrease in AIDS-related deaths and a 22% decline in new HIV infections in the country [3].

Despite the welcome improvements in the areas of HIV care outlined above, barriers persist in other areas of the national AIDS response in Sierra Leone. For example, the UNAIDS 2019 report cited earlier observed that there was uneven progress across the 90-90-90 targets. In 2018, fewer than half (49%) of PLWH in Sierra Leone knew their HIV status, and 41% of HIV-diagnosed patients were receiving ART, while only 26% of those on treatment were virally suppressed, the latter being likely due to a combination of poor treatment adherence and high prevalence of HIVDR in this setting [3]. In addition, recent studies by our group have observed other challenges in the local HIV care continuum, including high rates of late-stage HIV presentation [9], AIDS-associated in-hospital mortality [10], and high prevalence of HIV/HBV [11,12]. This is further compounded by the fact that as Sierra Leone and other LMICs have moved towards implementing integrase strand transfer inhibitor (INSTI)-based regimens as first-line ART, there is a dearth of country-specific studies to inform HIVDR prevention and management strategies due to limited laboratory testing capability.

In the first study characterizing HIVDR in Sierra Leone, we previously observed a pre-treatment HIVDR (PDR) prevalence of 37% (*n* = 64) and an acquired HIVDR (ADR) prevalence >95% (*n* = 151) among adult HIV-infected individuals aged >18 years who were receiving routine HIV care at Connaught Hospital, the country’s main referral health facility in Freetown [13]. Herein, we describe for the first time the prevalence of HIV-1 subtypes and resistance-associated mutations (RAMs) among children, adolescents, and pregnant women in Sierra Leone—three high-risk demographic groups that are disproportionately impacted by the HIV epidemic. 

## 2. Materials and Methods

### 2.1. Study Design, Populations, and Context

We conducted a cross-sectional study of 3 demographic groups of HIV-infected patients selected consecutively during February through May 2019, as follows: (1) children aged 2–9 years inclusive, (2) adolescents aged 10–18 years inclusive, and (3) pregnant women aged >18 years. The children and adolescents were enrolled at the HIV Clinic at Ola during Children’s Hospital, while the pregnant women were recruited at the HIV and Antenatal Clinic at the Princess Christian Maternity Hospital located in Freetown, Sierra Leone. Both hospitals serve as the national referral health centers for obstetric and pediatric cases and are major teaching affiliates of the College of Medicine and Allied Health Sciences of the University of Sierra Leone. The children and pregnant women were not biologically related.

Due to lack of HIVDR testing capability in Sierra Leone, none of the study participants had ever had baseline HIVDR testing at ART initiation; study participants were, therefore, selected consecutively using convenience sampling. At the time of study enrollment, the Sierra Leone National HIV Treatment Guidelines from 2017 (unpublished document from the National AIDS Control Programme of Sierra Leone) recommended a dual nucleos(t)ide reverse transcriptase inhibitor (NRTI) backbone, i.e., tenofovir/lamivudine (TDF/3TC), abacavir/lamivudine (ABC/3TC), or zidovudine/lamivudine (AZT/3TC) for plus the non-nucleotide reverse transcriptase inhibitors (NNRTIs) efavirenz (EFV) or nevirapine (NVP) as the preferred first-line regimen for all age groups. The ritonavir-boosted protease inhibitors (PIs) lopinavir (LPV/r) or atazanavir (ATV/r) were recommended as preferred second-line treatment for adults, or as alternative first-line for children <3 years who were exposed to nevirapine for prevention of MTCT.

### 2.2. Ethical Considerations

The study was conducted according to the guidelines of the Declaration of Helsinki and was approved by the Institutional Review Board of Case Western Reserve University/University Hospitals Cleveland Medical Center (approved 16 July 2019) and the Sierra Leone Ethics Scientific and Research Committee (approved 28 February 2019). Written informed consent was obtained from participants aged ≥15 years, whereas parental or guardian consent was obtained for participants <15 years.

### 2.3. Biosample Collection and Molecular Analysis

For each patient, 3 to 5 mL of venous blood were collected into tubes (BD Vacutainer^®^ K2 EDTA, Waltham, MA, USA) and centrifuged at 2000 g for 10 min. Plasma was collected into cryogenic vials (Nunc^®^ CryoTubes^®^, Waltham, MA, USA) and stored at −20 °C in Sierra Leone before transporting on dry ice (−80 °C) to the Galicia Sur Health Research Institute in Vigo, Spain, for molecular analysis. HIV reverse transcriptase (RT), protease (PR), and integrase (IN) were amplified from virus-derived plasma using an in-house Sanger sequencing protocol as previously described [13]. FASTA sequences were assembled using the Sequencer v5.4.6 DNA sequences analysis software (Gene Codes Corporation, Ann Arbor, MA, USA) and aligned with the reference sequence HXB2 (GenBank accession number K03455.1). HIV genetic subtypes and HIVDR mutations were identified and interpreted using the Stanford HIVDR database (freely available online at http://hivdb.stanford.edu, accessed on 21 June 2020). 

The genotypic susceptibility score (GSS), which estimates the number of HIV active drugs in a given combination ART regimen, was calculated as follows: (1) susceptible and potential low-level resistance were scored as 1, (2) low-level and intermediate resistance were pooled as intermediate and scored as 0.5, and high-level resistance was scored as 0. The GSS was assigned by summing the individual resistance scores for each first-line drug in the combination ART regimen as we previously described [13]. 

### 2.4. Statistical Analyses

Statistical analysis was performed using the Statistical Package for the Social Sciences software (SPSS 26.0, Chicago, IL, USA). Categorical variables were presented as frequency (percentages) and were compared using Pearson’s chi-squared test. Continuous variables were expressed as median (interquartile range, IQ; or range) and compared using the non-parametric Mann–Whitney U-test or Kruskal–Wallis test, where appropriate. *p* < 0.05 was considered statistically significant.

## 3. Results

### 3.1. Characteristics of the Study Population

A total of 197 PLWH were enrolled in the study, consisting of 96 children, 47 adolescents, and 54 pregnant women. Table 1 describes the main demographic and clinical characteristics of the three demographic groups. About half of children 51.0%, 49/96) and adolescents (53.2%, 25/47) were male, with median ages 5 years (IQR 4–7) and 13 years (IQR 10–15), respectively. The median CD4 counts were 985 (IQR 596–1407) cells/mm^3^ and 544 (IQR 260–849) cells/mm^3^, respectively, with 100% of children and 93.6% of adolescents having acquired HIV through MTCT. All children and adolescents (100%) were ART-experienced, with a median treatment duration of 36 (IQR 12–60) months and 84 (IQR 60–108) months, respectively. The majority of children (81.3%, 78/96) and adolescents (53.2%, 25/47) were on first-line ART.

For the pregnant women, the median age were 26 years (IQR 23–31) and the median CD4 count was 526 cells/mm3 (IQR 459–698). The majority (72.2%, 39/54) were ART-experienced with median ART duration of 3 months (range 1–96) and were receiving tenofovir/lamivudine/efavirenz, TDF/3TC/EFV (72.2%, 39/54). Over a quarter of the pregnant women (27.8%, 15/54) were new HIV diagnosis, with the majority of them (73.3%, 11/15) diagnosed during the second or third trimester of pregnancy. 

### 3.2. HIV Sequence Distributions by Demographic Groups

Plasma samples were analyzed from all 197 patients, and the HIV pol region was successfully sequenced in 108 patients, distributed as follows: 43 children, 33 adolescents, and 32 pregnant women. This yielded an overall sequencing success rate of 54.8% (108/197). The sequencing success rates by demographic groups were as follows: children, 44.8% (43/96); adolescents, 70.2% (33/47); and pregnant women, 59.3% (32/54).

### 3.3. HIV Genotypic Subtypes

The majority of patients were infected with the HIV-1 subtype CRF02_AG (87.9%, 95/108), followed by subtypes G (9.2%, 10/108), A (1.8%, 2/108), and C (0.9%, 1/108).

### 3.4. Prevalence of RAMS among Children and Adolescents

Genotypic resistance testing was performed on all 96 children (100%) and 47 adolescents (100%), which yielded 64 RT, 57 PR, and 67 IN sequences.

#### 3.4.1. NRTIs and NNRTIs

Nearly 80% (51/64) of RT sequences from children and adolescents harbored RAMs to NRTIs and NNRTIs. 

A total of 117 NRTI RAMs were identified from these two groups and were distributed as follows: as single RAMs (32.8%, *n* = 21/64), or in combinations of two (20.3%, *n* = 13/64), three (12.5%, *n* = 8/64), or five RAMs (9.4%, *n* = 6/64). On the other hand, 133 NNRTI RAMs were identified, either as a single RAM (18.8%, *n* = 12/64), or in combinations of two (18.8%, *n* = 12/64), or three (26.6%, *n* = 17/64) RAMs.

**Figure 1:**
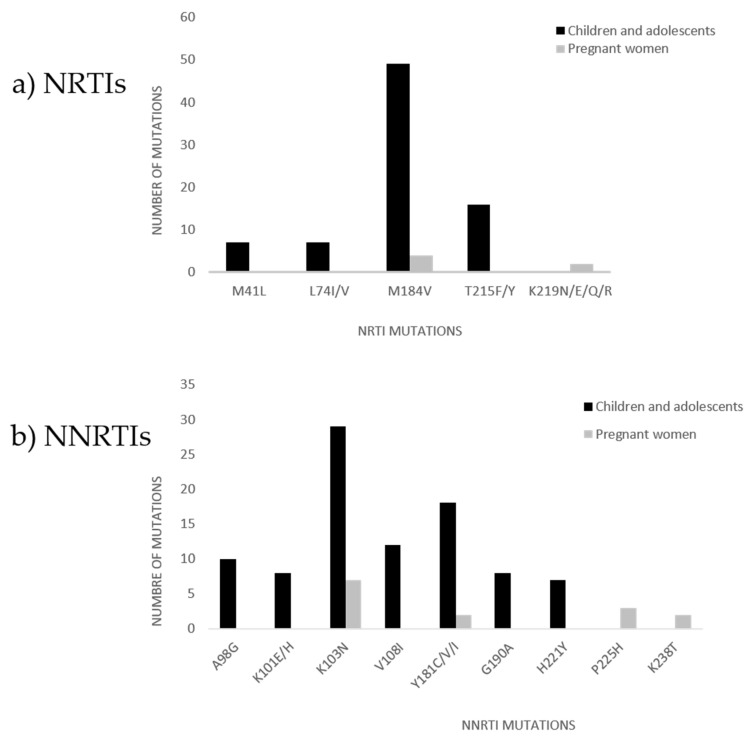
Common mutations to (**a**) NRTIs and (**b**) NNRTIs in children, adolescents, and pregnant women. Abbreviations: NRTI, nucleos(t)ide reverse transcriptase inhibitor; NNRTI, non-nucleotide reverse transcriptase inhibitor.

The most prevalent RT RAMs among children and adolescents and their relative proportions were as follows: M184V (76.6%, *n* = 49/64), K103N (45.3%, *n* = 29/64), Y181C/V/I (28.1%, *n* = 18/64), T215F/Y (25.0%, *n* = 16/64), V108I (18.8%, *n* = 12/64), A98G (15.6%, *n* = 10/64), G190A (12.5%, *n* = 8), K101E/H (12.5%, *n* = 8), M41L (10.9%, *n* = 7/64), L74I/V (10.9%, *n* = 7/64), and H221Y (10.9%, *n* = 7/64) (Figure 1a,b). 

#### 3.4.2. PIs and INSTIs

We observed 4 PI-associated RAMs in 6 children/adolescents, as follows: the combination mutation I54V + V82A (*n* = 2), M46I/M (*n* = 1), and I84I/V (*n* = 1). The combination mutation I54V+V82A confers intermediate resistance to all the currently approved PIs with the exception of darunavir, which retains full activity. Interestingly, there was no documented record of prior exposure to PI-based ART in these 6 patients.

Polymorphic accessory INSTI-selected mutations, which have minimal effect on INSTI susceptibility were observed in 8 children/adolescents, as follows: T97A (*n* = 2), E157Q (*n* = 5), and S230SR (*n* = 1).

#### 3.4.3. Genotypic Susceptibility Scores

Based on the GSS estimates, 76.3% had GSS < 3 to the WHO preferred first-line DTG-based ART, whereas 66.6% to 70% of the children had a GSS < 3 to the WHO preferred/alternative second-line PI-based regimens, respectively, i.e., they harbored genotypes that were not fully susceptible to all three antiretroviral drugs in the regimen (Table 2). Similarly, 87.5% and 92.8% to 100% of adolescents harbored genotypes that were not susceptible to the WHO preferred first-line and second-line regimens, respectively (Table 2).

### 3.5. Prevalence of RAMS among Pregnant Women

Genotypic resistance testing was performed on all 54 pregnant women (100%). A total of 34 RT, 27 PR, and 33 IN sequences were obtained, which were distributed as follows based on treatment status: (1) 27 RT, 21 PR, and 24 IN from ART-experienced patients (*n* = 39); and (2) 7 RT, 6 PR, and 9 IN from the newly diagnosed ART-naïve patients (*n* = 15). 

#### 3.5.1. NRTIs and NNRTIs

Overall, 76.5% (*n* = 26/34) of RT sequences harbored RAMs to NRTIs or NNRIs in pregnant women, which were distributed as follows: (1) ART-experienced (59.3%, *n* = 16/27), and (2) newly diagnosed ART-naïve (71.4%, *n* = 5/7). 

A total of 9 NRTI RAMs were identified, as follows: (1) 7 RAMs in the ART-experienced group, which occurred as a single mutation (2.6%, *n* = 1/39) or as a combination of two mutations (7.7%, *n* = 3/39); and (2) 2 RAMs in the newly diagnosed ART-naïve, which occurred as a combination of two mutations (28.6%, *n* = 2/7). On the other hand, 18 NNRTI RAMs were identified, as follows: (1) 5 RAMs in the newly diagnosed ART-naïve patients, which occurred as a combination of two (14.3%, *n* = 1/7) or three mutations (14.3%, *n* = 1/7); and (2) 13 RAMs in the ART-experienced patients, which occurred as a single mutation (7.4%, *n* = 2/27) or in combinations of two (7.4%, *n* = 2/27), three (3.7%, *n* = 1/20) or four mutations (3.7%, *n* = 1/27). 

The most prevalent RT RAMs in pregnant women were as follows: K103N (20.6%, *n* = 7/34), M184V (11.8%, *n* = 4/34), Y181C/V/I (5.9%, *n* = 2/34), P225H (8.8%, *n* = 3/34), K219N/E/Q/R (5.9%, *n* = 2/34), and K238T (5.9%, *n* = 2/34) (Figure 1a,b). 

#### 3.5.2. PIs and INSTIs

No PIs RAMs were observed, whereas three accessory INSTI-selected mutations T97A (*n* = 1), E157Q (*n* = 2), and G163R (*n* = 1), which have minimal effect on INSTI susceptibility, were observed in four pregnant women.

#### 3.5.3. Genotypic Susceptibility Scores

Based on GSS distributions, 13.6% of the pregnant women had GSS < 3 to the WHO preferred first-line DTG-based ART, whereas 66.6% to 100% of the pregnant women had a GSS < 3 to the WHO preferred/alternative second-line PI-based regimens, respectively, i.e., they harbored genotypes that were not fully susceptible to all three antiretroviral drugs in the regimen (Table 2).

## 4. Discussion

This is the first study from Sierra Leone to characterize HIV-1 genetic subtypes and HIVDR among children, adolescents, and pregnant women. In this cross-sectional survey, we assessed the prevalence of RAMs in a cohort of 96 children aged 2–9 years, 47 adolescents aged 10–18 years, and 54 pregnant women >18 years at the two main referral children’s and women’s health facilities, respectively, in Freetown. We observed a high prevalence of RAMs to NRTIs (up to 77% in children and adolescents) and NNRTIs (nearly 50%) in the three demographic groups, whereas the prevalence of RAMs to PIs and INSTIs was relatively low or absent. Our findings confirm the situation previously described in the WHO 2019 HIVDR report [1], as well as other recent studies from the West Africa region, including our previous study describing HIVDR among adult PLWH in Sierra Leone [13,14,15,16,17,18]. 

Current international ART treatment guidelines recommend a dual NRTI-backbone in combination with an INSTI or a boosted-PI with a high genetic barrier to developing resistance such as darunavir/ritonavir (DRV/r) in the treatment of HIV-infected individuals [12,19,20,21]. Sierra Leone is an INSTI-naïve stetting, as INSTI-based ART was only recently introduced in 2021. Despite this, we found several polymorphic accessory INSTI-selected mutations (e.g., T97A and E157Q) in eight children/adolescents and four pregnant women associated with low-level drug resistance. Reassuringly, however, we did not observe any clinically relevant INSTI RAMs in this study or in our previous HIVDR study from Sierra Leone. These findings corroborate current evidence, derived largely from studies in B subtypes, which suggests that globally, the prevalence of major INSTI RAMs to DTG is very low among PLWH without prior exposure to DTG [22,23,24], supporting the notion that DTG may be safe to use in this INSTI-naïve setting. 

Despite its high potency, good tolerability, and high genetic barrier to developing drug resistance, safety concerns persist around the peri-conception use of DTG in women of child-bearing age and during the first trimester of pregnancy, due to the reported higher incidence of neural tube defects [25,26,27]. Another point of caution that has been raised in relation to the roll-out of DTG in SSA is that, due to insufficient studies on the subject, it is uncertain what impact, if any, circulating HIV non-B subtypes in SSA will have on the efficacy of DTG. Several studies have observed a higher frequency of INSTI-associated polymorphisms among non-B subtypes [28,29,30,31]. However, current experience with DTG and other INSTIs have shown high rates of virologic suppression (>90%) in non-B subtypes, including CRF02_AG, the dominant HIV subtype circulating in Sierra Leone and in West and Central Africa [32,33,34,35]. Notwithstanding, this is likely to be an area of careful investigation in the future, as DTG becomes more widely used in this region, which has the most diverse genetic subtypes.

We found that M184V was the most frequent NRTI RAM, occurring with a prevalence of nearly 77% among children/adolescents and 11% of pregnant women. This mutation is selected by 3TC and emtricitabine (FTC), which significantly reduces their activity (by about 100- to 1000-fold, respectively) while conferring low-level resistance to ABC [36,37,38]. In contrast, M184V induces hypersusceptibility to TDF and AZT, while simultaneously limiting the emergence of drug resistance to both agents [39,40,41]. This is particularly relevant to the Sierra Leone context, given that the most commonly used NRTI in clinical care in Sierra Leone is 3TC, with 100% of our ART-experienced study participants having been exposed to it. In the adult study participants, TDF was the other commonly used NRTI, while in children and adolescents, either ABC or AZT constituted the second NTRI option. Taken all together, these findings may have two important clinical implications. Firstly, patients failing first-line ART based on the 2017 ART guidelines in Sierra Leone have limited options for second-line therapy, as estimated by the GSS (Table 2). Secondly, with high rates of HIV/HBV coinfection in this setting (up to 22% based on previous studies by our group) [11,12]—a large proportion of patients on 3TC-based and/or TDF-sparing ART regimens are likely to be receiving suboptimal treatment for both HIV and HBV infections, heightening their risk for progression to AIDS, liver cirrhosis, and end-stage liver disease. Thus, screening for HBV in all HIV-infected patients before ART initiation and at the time of switching ART is essential to guiding optimal ART choice.

The marked disparity in frequency of M184V-selected viruses among children and adolescents (77%) and pregnant women (11%) was noteworthy. Studies have shown that M184V is associated with reduced replicative activity in the absence of drug pressure, compared with the wild-type (WT) virus [42,43,44]. The children and adolescents were all (100%) ART-experienced for a much longer duration (median of 48 months) compared with the pregnant women (72.2% ART-experienced for a median duration of 3 months) and would, therefore, be expected to accumulate more M184V viruses, due to the overall higher drug exposure. We hypothesize that this finding may also have been driven, in part, by differing levels of medication adherence in the three study groups. It is plausible that while adherence may have been low in both groups, it was comparatively much lower among pregnant women, resulting in less pharmacologic pressure in this group and increasing the reversion rate of M184V-selected viruses to WT viruses. We were unable to assess drug adherence to confirm this hypothesis. Poor ART adherence remains a widespread global challenge, and effective strategies to address this problem will help in limiting drug resistance, maximize the benefits of ART, and improve treatment outcomes.

The prevalence of the NNRTI mutation K103N, which is selected by EFV and NVP was high in all three study groups. This finding is in line with reports from the SSA, where the frequency of K103 has exceeded 10% in most LMICs reporting HIVDR surveillance data to the WHO [1]. In this study, the prevalence of K103 (was much lower (i.e., 45.3% in children/adolescents and 20.6% in pregnant women) than we previously described among the adult population receiving ART in Sierra Leone [13]. There are limited studies on MTCT rates in Sierra Leone; however, according to programmatic data from 2016, PMTCT ART coverage was 67.3% [45] and there was a decline in MTCT rates from 22% (2011) to 13% (2014) countrywide [8]. Despite this, nearly 75% of pregnant women who were newly diagnosed with HIV in this study were diagnosed in the second or third trimesters. If this observation is reflective of broader trends, the combination of the high prevalence of RAMs to NVP (the principal drug used in PMTCT treatment in Sierra Leone) and late diagnosis of HIV during pregnancy may have far-reaching policy implications for the success of PMTCT efforts in Sierra Leone and LMICs with similar MTCT characteristics. 

Our study had several limitations. Firstly, our study enrolled a small sample size from a single clinical site, which limits the generalizability of study findings. Secondly, although the issues of pretreatment (PDR) and/or transmitted drug HIV drug resistance (TDR) remain important barriers to reducing vertical HIV transmission, we were unable to assess this fully due to the relatively small number of ART-naïve pregnant study population. Thirdly, due to insufficient plasma samples, VL measurement could not be performed for all patients. Finally, because the majority of the patients were on ART for a long period of time (median period 36 months and 84 months in children and adolescents, respectively), we suspect that a substantial proportion of these patients were either virologically suppressed or had low viremia, which may have contributed to the low rate of sequencing success, especially among children. Nonetheless, our findings contribute to our current understanding of circulating HIV strains and HIVDR among three vulnerable populations that are disproportionately affected by the growing HIV epidemic and may have some policy implications for PMTCT program activities. Larger studies are needed to assess the countrywide HIVDR prevalence rates and associated factors to help inform treatment strategies and public health approaches in the national AIDS response in Sierra Leone.

## 5. Conclusions

We observed a high prevalence of circulating RAMs to NRTIs (especially 3TC) and NNRTIs (EFV and NVP) among HIV-infected children, adolescents, and pregnant women in Freetown, Sierra Leone. In contrast, the prevalence of RAMs to PIs and INSTI was low or absent. Although the three populations were not biologically related, our findings may have implications for the success of PMTCT efforts in the country. Furthermore, our findings suggest the need for a national HIVDR surveillance program to help guide ART choice and improve treatment outcomes for patients.

## Figures and Tables

**Table 1:** Baseline characteristics of the study population.

Characteristics	Children N = 96	Adolescents N = 47	Pregnant Women N = 54
**Gender**			
Male	49 (51.0)	25 (53.2)	-
Female	47 (49.0)	22 (46.8)	54 (100)
**Age, years**			
Median (IQR)	5 (4–7)	13 (10–15)	26 (23–31)
2–5	50 (52.1)	-	-
6–9	46 (47.9)	-	-
10–18	-	47 (100)	-
18–30	-	-	36 (75.0)
31–40	-	-	12 (25.0)
**Mode of HIV acquisition**			
Mother to child transmission	96 (100)	44 (93.6)	-
Sexually transmitted	-	3 (6.4)	-
Median duration of pregnancy (IQR), weeks	-	-	22 (16–29)
**Trimester of newly diagnosed** N = 15 (100%)			
First (1–13 weeks)	-	-	4 (26.7)
Second (14–26 weeks)	-	-	8 (53.3)
Third (27+ weeks)	-	-	3 (20.0)
CD4 count, median (IQR), cells/mm^3^	985 (596–1407)	544 (260–849)	526 (459–698)
**Current ART regimens**			
TDF+ 3TC+ EFV	-	7 (14.9)	39 (72.2)
AZT+ 3TC+ EFV	7 (7.3)	4 (8.5)	-
AZT+ 3TC+ NVP	25 (26.0)	20 (42.6)	-
AZT+ 3TC+ LPV/r	6 (6.3)	-	-
ABC+ 3TC+ EFV	27 (28.1)	9 (19.1)	-
ABC+ 3TC+ NVP	15 (15.6)	2 (4.3)	-
ABC+ 3TC+ LPV/r	16 (16.7)	5 (10.6)	-
Not on ART	-	-	15 (27.8)
**Type of ART**			
First-line *	78 (81.3)	25 (53.2)	39 (72.2)
Second-line **	18 (18.8)	22 (46.8)	-
**Duration of ART exposure, months**			
Median (IQR)	36 (12–60)	84 (60–108)	3 (1–9)
≤24	42 (43.8)	8 (17.0)	39 (72.2)
25–48	23 (24.0)	2 (4.3)	-
49–60	23 (24.0)	12 (25.5)	-
>60	6 (8.4)	25 53.2)	-

Abbreviations: ART, antiretroviral therapy; IQR, interquartile range. * First-line and ** Second-line regimens based on the 2017 Sierra Leone HIV treatment guidelines (unpublished document from the National AIDS Control Programme of Sierra Leone); -, not applicable; bold distinguishes between variables with sub-categories

**Table 2:** Genotypic scores to the preferred first-line and second-line ART based on the 2018 WHO guidelines.

Population	Preferred WHO First-Line Regimens	GSS < 3 *n* (%)	Preferred or Alternative WHO Second-Line Regimens	GSS < 3 *n* (%)
Children	AZT + 3TC + DTG	29 (76.3)	AZT + 3TC + LPV/r (or ATV/r)	24 (68.6)
	ABC + 3TC + DTG	29 (76.3)	AZT + 3TC + DRV/r	24 (68.6)
			ABC + 3TC + LPV/r (or ATV/r)	24 (68.6)
			ABC + 3TC + DRV/r	6 (66.6)
			ABC + 3TC + RAL	7 (70.0)
Adolescents	AZT + 3TC + DTG	14 (87.5)	AZT + 3TC + LPV/r (or ATZ/r)	13 (92.8)
	ABC + 3TC + DTG	14 (87.5)	AZT + 3TC + DRV/r	13 (92.8)
	TDF + 3TC + DTG	14 (87.5)	ABC + 3TC + LPV/r (or ATZ/r)	24 (68.6)
			ABC + 3TC + DRV/r	6 (100)
Pregnant women	TDF + 3TC (or FTC) + DTG	3 (13.6)	TDF + 3TC (or FTC) + ATV/r	3 (17.6)
	ABC + 3TC + DTG	3 (13.6)	TDF + 3TC (or FTC) + LPV/r	3 (17.6)
	AZT + 3TC + DTG	3 (13.6)	TDF + 3TC (or FTC) + DRV/r	3 (17.6)
			AZT + 3TC + ATV/r	3 (17.6)
			AZT + 3TC + LPV/r	3 (17.6)
			AZT + 3TC + DRV/r	3 (17.6)

Abbreviations: ART, antiretroviral therapy; GSS, genotypic susceptibility scores.

## Data Availability

The raw de-identified data supporting the conclusions of this article will be made available by the authors, without undue reservation, to any qualified researcher.
